# Microwave Liquefaction of Crystallized Jordanian Honeys and Its Impact on Quality Parameters

**DOI:** 10.1021/acsomega.5c11767

**Published:** 2026-03-25

**Authors:** Abd Almajeed Al-Ghzawi, Shahera Zaitoun, Taha Rababah, Nezar Samarah, Akram Bin Zaidan, Sevil Yücel, Huda Fish

**Affiliations:** † Department of Plant Production, Faculty of Agriculture, 37251 Jordan University of Science and Technology, P.O. Box: 3030, Irbid 2211, Jordan; ‡ Department of Plant Production & Protection, Faculty of Agricultural Technology, 59303 Al-Balqa Applied University, P.O. Box 206, Salt 19117, Jordan; § Department of Nutrition and Food Technology, Faculty of Agriculture, Jordan University of Science and Technology, P.O. Box: 3030, Irbid 2211, Jordan; ∥ Department of Bioengineering, Faculty of Chemical and Metallurgical Engineering, Yildiz Technical University, 34220, Istanbul, Türkiye; ⊥ College of Agriculture, University of Al Dhaid, Al Dhaid, Sharjah 12600, United Arab Emirates; # School of Biological Science, Queen’s University Belfast, 19 Chlorine Gardens, Belfast, Northern Ireland BT9 5DL, U.K.

## Abstract

Crystallization (granulation) is a common and commercially undesirable phenomenon in honey, as consumers generally prefer liquid products. Conventional thermal treatments can liquefy crystallized honey, but are often associated with deterioration of quality sensitive attributes. In this study, microwave liquefaction was evaluated as an alternative to conventional water bath heating (65 °C for 25 min), and its effects on the quality of three Jordanian unifloral honeys (*Centaurea*, *Ballota*, and *Echinops*) were assessed. Crystallized honeys were subjected to microwave treatment at three nominal power levels (30, 60, and 90%) using systematically optimized power and time combinations specific to each botanical origin, and the outcomes were compared with conventional heating. Microwave processing achieved complete crystal dissolution in all honey types, with liquefaction time decreasing as microwave power increased and varying according to botanical origin, whereas conventional heating resulted in incomplete crystal removal under the applied conditions. Both processing methods caused moderate reductions in moisture content, pH, total phenolic content, and enzymatic activities (diastase and invertase). However, these changes were consistently less pronounced following microwave treatment. Total soluble solids, electrical conductivity, and sugar composition were largely unaffected by either method. Although hydroxymethylfurfural content increased after thermal processing, all values remained within Codex specified limits. Overall, the results demonstrate that botanical origin dependent optimization of microwave liquefaction enables rapid and effective decrystallization while better preserving key quality attributes compared with prolonged conventional heating. These findings support microwave processing as a controllable alternative for honey liquefaction within regulatory limits, while emphasizing the need for honey specific optimization to ensure consistent quality preservation across different botanical origins.

## Graphical abstract

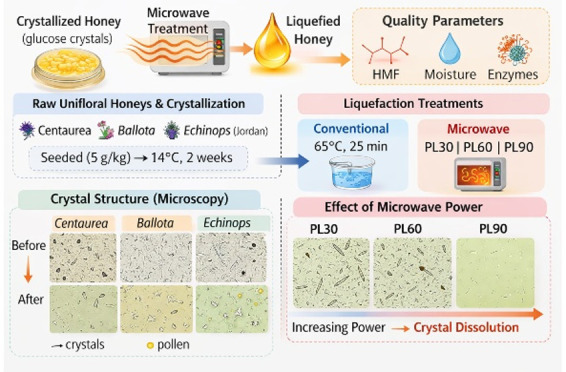

## 1 Introduction

Honey is a naturally occurring carbohydrate rich food produced by *Apis mellifera* bees from floral nectar or honeydew secretions, followed by enzymatic modification, dehydration, and maturation within the honeycomb.^1,2^ Global consumer demand for honey continues to grow, driven by its perceived nutritional value, functional properties, and preference for minimally processed foods.^3^

From a compositional perspective, honey consists predominantly of carbohydrates, with fructose and glucose accounting for approximately 60–85% of total sugars, alongside smaller proportions of disaccharides, oligosaccharides, organic acids, proteins, enzymes, phenolic compounds, and minerals.^4,5^ Variations in floral origin, climate, and postharvest handling significantly influence honey composition and, consequently, its physicochemical behavior. In particular, the glucose to fructose ratio and moisture content govern viscosity, hygroscopicity, and the propensity for crystallization during storage.^6^

Fresh honey is typically liquid; however, crystallization (granulation) is a spontaneous and thermodynamically driven process that occurs over time, depending on botanical origin, temperature, and water content.^7,8^ Although crystallization does not compromise safety, it is widely perceived as undesirable by consumers due to its impact on texture, spreadability, and visual appearance. To delay crystallization and improve shelf stability, honey is commonly subjected to thermal processing prior to packaging. Conventional treatments typically involve prolonged heating (e.g., 57 °C for 1 h or ≥60 °C for 30 min), sufficient to inactivate osmophilic yeasts and retard granulation, while industrial flash processes may reach 70–77 °C for short durations.^5,9,10^

Despite their effectiveness, conventional thermal treatments are associated with well documented quality trade offs, including reductions in enzymatic activity (particularly diastase and invertase), degradation of heat sensitive bioactive compounds, and increased formation of hydroxymethylfurfural (HMF), a key indicator of excessive heating and quality deterioration.^5,11^ Regulatory standards, therefore, impose strict limits on these parameters, constraining the intensity and duration of acceptable thermal processing. The reliance on extended heating times under conventional conditions highlights the need for alternative approaches that can achieve rapid liquefaction of crystallized honey while minimizing quality losses. Recent studies comparing thermal liquefaction strategies have further demonstrated that processing intensity and duration critically influence enzyme activity, phenolic retention, and hydroxymethylfurfural (HMF) formation, underlining the need for controlled, rapid liquefaction approaches tailored to honey type.^12^

Microwave heating has been proposed as a non conventional thermal technology capable of addressing these limitations. Unlike conductive heating, microwave processing enables volumetric energy absorption, leading to rapid and relatively uniform temperature increases with substantially reduced processing times. Previous studies have demonstrated the potential of microwave treatment for honey liquefaction, microbial reduction, and delay of recrystallization, with variable effects on physicochemical and biochemical quality attributes depending on power level, exposure time, and honey type.^13^ However, despite these promising findings, important knowledge gaps remain. Most existing studies evaluate a single honey type, fixed microwave conditions, or a limited subset of quality parameters, thereby limiting broader interpretation and industrial transferability. In particular, it remains insufficiently resolved how botanical origin influences optimal microwave power–time regimes and the balance between effective decrystallization and preservation of enzymatic and bioactive quality attributes under controlled comparative conditions. The present study addresses this gap by systematically optimizing microwave power–time treatments across three botanically distinct monofloral honeys and integrating physicochemical, enzymatic, bioactive, and regulatory quality indicators within a unified experimental framework. By directly benchmarking microwave processing against conventional water bath heating, this work aims to clarify both honey specific responses and general processing trends relevant to industrial applications.

In this context, the present study investigates microwave processing as an alternative to conventional water bath heating for the liquefaction of crystallized Jordanian honeys of different botanical origins. The scientific novelty of this work lies in the systematic optimization of microwave power and time regimes across three botanically distinct unifloral honeys within a unified experimental framework, coupled with a comprehensive evaluation of physicochemical, enzymatic, bioactive, and regulatory quality indicators.

Specifically, the study aims to (i) determine the optimal combinations of microwave power and exposure time required for efficient crystal dissolution in *Centaurea*, *Ballota*, and *Echinops* honeys; (ii) compare the effectiveness of microwave and conventional treatments in achieving complete liquefaction; and (iii) evaluate the impact of both treatments on key physicochemical and quality parameters, including moisture content, total soluble solids, pH, color, electrical conductivity, HMF formation, total phenolic content, antioxidant activity, diastase and invertase activities, and sugar composition. By explicitly linking liquefaction efficiency, quality preservation, and regulatory compliance, this study provides practical insights into the applicability and limitations of microwave assisted liquefaction for honey processing.

## 2 Materials and Methods

### 2.1 Honey Samples and Authentication

Three unifloral raw honeys (*Centaurea*, *Ballota*, and *Echinops*) were obtained from a local producer (Brothers Co., Irbid, Jordan). All samples were harvested during the same production season to reduce variability related to climatic or seasonal factors. Sample authenticity and floral origin were verified by pollen sediment analysis conducted at the Beekeeping Laboratory, Faculty of Agriculture, Jordan University of Science and Technology, following established melissopalynological procedures.^14,15^ Honey samples were stored at ambient temperature (20–22 °C) for no longer than 2 weeks prior to experimentation to minimize physicochemical changes prior to processing. All treated and untreated samples were derived from the same bulk batches for each honey type to minimize compositional variability and ensure valid comparison between liquefaction treatments.

### 2.2 Induced Crystallization

To obtain uniformly crystallized samples, liquid honey was seeded with crystallized honey (5 g per 1 kg of honey), mixed thoroughly, and stored at 14 °C for 2 weeks until complete crystallization was achieved. The seed crystals were obtained from the corresponding honey type to avoid compositional mismatch and ensure crystallization behavior representative of each botanical origin. This procedure was applied identically to all honey types under controlled temperature conditions to ensure reproducibility and comparability across treatments.

### 2.3 Liquefaction Treatments

#### 2.3.1 Conventional Thermal Treatment

Crystallized honey samples (150 g) were transferred into 150 mL glass jars, covered with aluminum foil, and heated in a thermostatically controlled water bath at 65 °C for 25 min with continuous stirring, following conventional thermal processing approaches described previously.^16^ The selected temperature–time combination reflects commonly applied industrial liquefaction conditions intended to ensure crystal melting while limiting excessive thermal degradation. After heating, samples were rapidly cooled to room temperature and stored under ambient conditions prior to analysis.

#### 2.3.2 Microwave Treatment

Microwave liquefaction was performed using a laboratory microwave oven (Samsung, Korea) with a maximum output power of 850 W. Crystallized honey samples (100 g) were placed in 150 mL glass jars and treated individually at nominal power levels of 30%, 60%, and 90% of maximum output, corresponding to approximately 255, 510, and 765 W, respectively. These power levels were selected to span low, intermediate, and high microwave energy inputs relevant to laboratory scale honey processing. Samples were exposed for varying durations (30, 60, 90, and 120 s) depending on the honey type until complete liquefaction was achieved.

All treatments were conducted with the sample positioned centrally on the rotating turntable. Samples were intermittently stirred during heating to improve thermal distribution and reduce the likelihood of localized overheating. Fixed sample mass, identical container geometry, and consistent central positioning were maintained across all treatments to enhance experimental comparability. Although microwave heating is known to generate non uniform thermal fields, the combination of rotational motion and intermittent stirring was implemented as a practical control strategy to mitigate potential temperature gradients under laboratory scale conditions.

Microwave power was defined in absolute terms (255, 510, and 765 W), and all treatments were conducted using fixed sample mass, container geometry, and central positioning on a rotating turntable, with intermittent stirring to improve thermal uniformity. The sample temperature was measured immediately after treatment and recorded as peak temperature. Although continuous in situ temperature monitoring or spatial thermal mapping was not performed, the combination of standardized sample configuration, rotational motion, and intermittent stirring was implemented to enhance experimental control under laboratory scale conditions. Following microwave exposure, samples were cooled rapidly under running tap water to prevent further thermal degradation and quality deterioration and then stored at ambient temperature until analysis.^17^

We acknowledge that microwave heating may result in localized temperature gradients (“hot spots”), which represent a known limitation of the technology. Therefore, the recorded temperatures reflect end point measurements rather than real time spatial thermal distributions within the sample matrix. However, the consistent trends observed across quality indicators and the direct benchmarking against conventional water bath heating under controlled conditions support the validity of the comparative conclusions. The present findings should be interpreted as laboratory-scale optimization trends rather than definitive large-scale process uniformity. Future studies incorporating real time temperature monitoring or thermal imaging would further strengthen process control and scalability assessment.

### 2.4 Analytical Methods

A schematic overview of the experimental workflow, from crystallization through treatment and analysis, is provided in Figure 1 to enhance methodological transparency and reproducibility.

**Figure 1:**
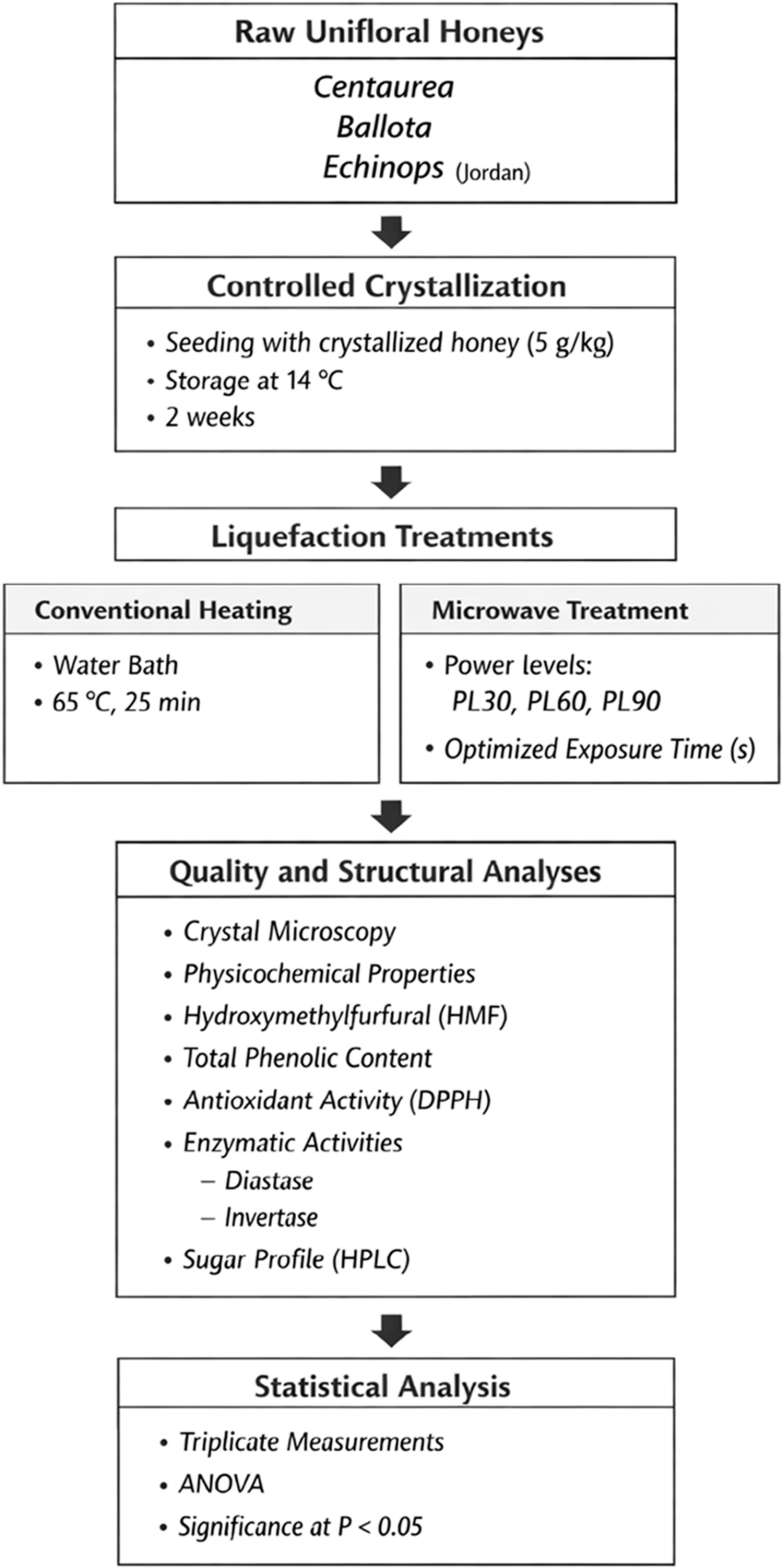
Experimental Workflow Schematic.

#### 2.4.1 Pollen Sediment Analysis

Sediment content was determined following standard melissopalynological procedures^14,15^ to confirm floral origin and sample authenticity prior to processing. Raw and treated honey samples (10 g) were dissolved in warm distilled water (40 °C), centrifuged, and the resulting sediments were mounted on microscope slides using glycerin gelatin. Pollen types were identified and quantified microscopically, and classification was based on relative pollen frequency, defined as very frequent (>45%), frequent (16–45%), rare (3–15%), or sporadic (<3%). This analysis was used for authentication purposes only and not for post treatment quality evaluation, ensuring that subsequent physicochemical and biochemical analyses reflected processing effects rather than botanical misclassification.

#### 2.4.2 Crystal Observation

Crystal morphology and presence were examined using an optical microscope (Optika C–P6, Italy) equipped with a digital camera (Optikam Pro6). A drop of honey was placed on a glass slide and examined at 400× magnification under ambient laboratory conditions. Representative images were captured for each treatment.^18^ Microscopic observation was used to qualitatively assess crystal presence, size, and morphology before and after liquefaction treatments, allowing visual comparison of crystallization state across honey types and processing conditions. Complete liquefaction was operationally defined as the absence of visible crystalline structures under microscopic examination, consistent with previous honey liquefaction studies. Microscopy was employed as a qualitative supportive tool rather than a quantitative image analysis method, and conclusions regarding liquefaction efficiency were interpreted in conjunction with physicochemical and compositional data (Sections 3.2–3.7).

#### 2.4.3 Physicochemical Parameters

Moisture content and total soluble solids (TSS) were determined using a temperature compensated hand-held refractometer at 20 °C, following standard procedures.^19^ Measurements were performed in triplicate for each sample, and mean values were reported.

pH values were measured in honey solutions (10 g honey dissolved in 75 mL deionized water) using a calibrated pH meter (Elmetron-Poland), with calibration performed using standard buffer solutions prior to analysis.^19^

Electrical conductivity was determined in honey solutions (20 g honey in 100 mL deionized water) and expressed as mS/cm at 20 °C, in accordance with international recommendations.^20^ EC measurements were used as an indicator of mineral content and botanical origin consistency across treatments.

Color was measured spectrophotometrically at 560 nm using a UV–vis spectrophotometer (Shimadzu, Japan) and expressed in mm Pfund according to USDA standards. Color analysis was included to assess potential thermal darkening associated with processing intensity.^21–24^

All physicochemical measurements were conducted on untreated, conventionally heated, and microwave treated samples under identical analytical conditions to ensure comparability.

#### 2.4.4 Hydroxymethylfurfural (HMF)

HMF content was quantified by HPLC following the method described by Pascual-Maté.^14^ Honey solutions were prepared under controlled conditions, filtered through 0.45 μm membrane filters and analyzed using a reverse phase column with UV detection at 285 nm. Quantification was performed using external calibration with HMF standards, and results were expressed as mg/kg honey. All measurements were conducted in triplicate, and mean values were used for statistical analysis.

HMF was selected as a key chemical quality marker to assess the thermal impact of conventional and microwave treatments and to evaluate compliance with international honey quality regulations.

#### 2.4.5 Total Phenolic Content and Antioxidant Activity

Total phenolic content (TPC) was determined using the Folin–Ciocalteu method and expressed as mg gallic acid equivalents (GAE)/100 g honey, following established protocols.^15,16^ Gallic acid was used for external calibration, and absorbance was measured spectrophotometrically.

Antioxidant activity was evaluated using the 2,2-diphenyl-1-picrylhydrazyl (DPPH) radical scavenging assay and expressed as percentage inhibition, as described previously.^17^ All measurements were performed in triplicate, and mean values were used for statistical analysis. These assays were selected to assess the impact of liquefaction treatments on honey bioactive properties and to support comparison between microwave-assisted and conventional thermal processing.

#### 2.4.6 Enzymatic Activities

Diastase activity was measured according to the Schade method as described by AOAC and Bogdanov and expressed as diastase number (DN).^19,25^ Invertase activity was determined using the *p*-nitrophenyl-α-d-glucopyranoside (PNPG) method and expressed as invertase number (IN).^19^ Both enzymatic assays were performed in triplicate for each treatment and honey type. Enzyme activities were used as sensitive indicators of thermal impact and quality preservation during liquefaction, given their well documented heat susceptibility and regulatory relevance in honey quality assessment.

#### 2.4.7 Sugar Profile

Sugar composition (fructose, glucose, sucrose, maltose) was determined by high performance liquid chromatography (HPLC) with refractive index detection using a carbohydrate analysis column, following established methods.^19^ Results were expressed as g/100 g honey. All analyses were performed in triplicate for each treatment and honey type. Sugar profiling was included to assess potential thermal degradation of reducing sugars and to verify compliance with international honey quality standards, particularly the sum of fructose and glucose following liquefaction treatments.

### 2.5 Statistical Analysis

All analyses were performed in triplicate. Results are expressed as mean ± standard deviation. Statistical comparisons were conducted using one way analysis of variance (ANOVA), with significance accepted at *P* < 0.05. Statistical analyses were performed using SAS software. Where appropriate, ANOVA was applied independently for each honey type to evaluate the effect of liquefaction treatment. Statistical significance was interpreted alongside the magnitude and practical relevance of observed changes to distinguish technological relevance from purely statistical effects.

## 3 Results and Discussion

### 3.1 Botanical Authentication by Pollen Spectrum

The pollen spectrum confirmed the unifloral authenticity and botanical classification of the three honey samples used in this study (Table 1). Honey sample #5 was dominated by *Centaurea iberica* pollen (58%), with *Sinapis arvensis* (18%) and *Silybum marianum* (12%) as secondary taxa, exceeding the threshold required for dominant pollen classification and confirming its designation as Centaurea honey.

**Table 1:**
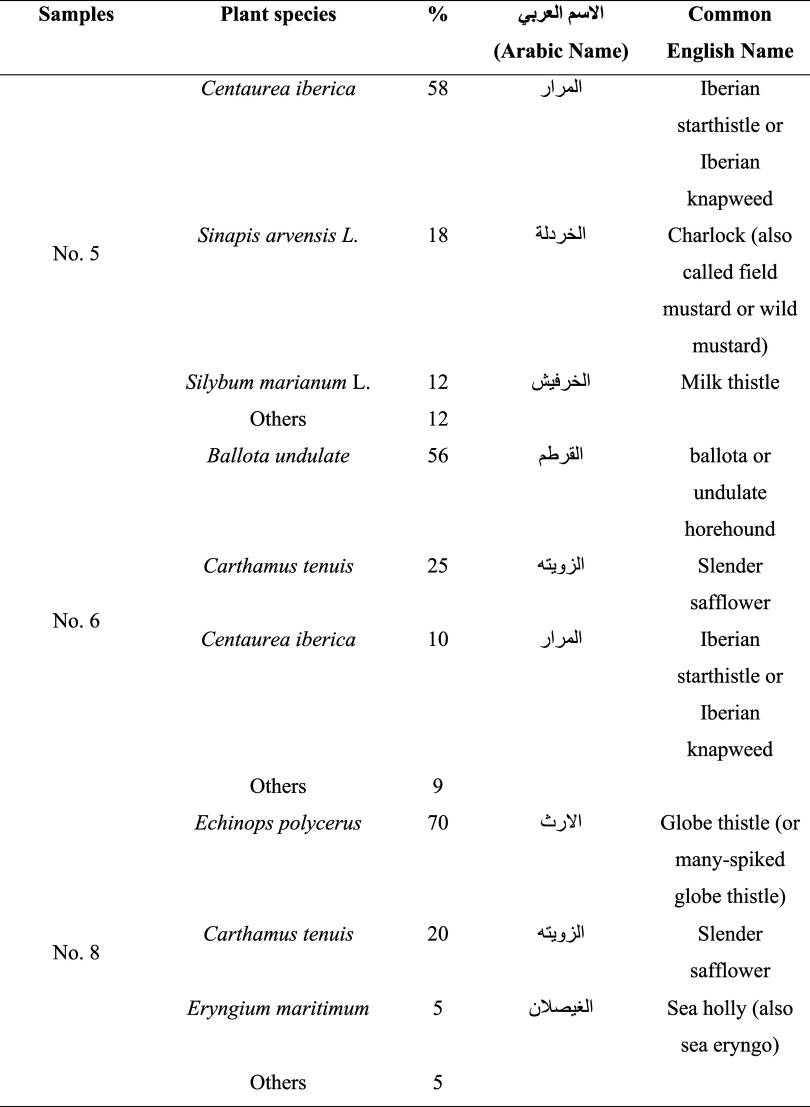
Classification of Honey Types According to Sediment Contents

Honey sample #6 exhibited dominant *Ballota undulata* pollen (56%), followed by *Calamus tenuis* (25%) and *C. iberica* (10%), supporting its classification as Ballota honey. Honey sample #8 was characterized by *E. polycerus* pollen dominance (70%), with *C. tenuis* (20%) and *Eryngium maritimum* (5%), clearly establishing its unifloral identity as Echinops honey. Minor amounts of indeterminate pollen were detected in all samples, which is typical of natural honeys and does not compromise unifloral designation.

Confirmation of botanical origin is essential for interpreting differences in crystallization behavior, microwave liquefaction efficiency, and quality preservation discussed in subsequent sections, as floral source influences sugar composition, moisture content, and thermal response.

### 3.2 Liquefaction Performance and Crystal Disappearance

#### 3.2.1 Crystal Morphology before Treatment

Microscopic examination of untreated crystallized honeys revealed dense and well developed crystal networks with clear differences in crystal size, shape, and packing density among the three botanical origins (Figure 2). Predominantly needle like and irregular crystalline structures were observed, consistent with glucose monohydrate crystallization in a supersaturated honey matrix. Variations in crystal morphology among the honeys likely reflect differences in sugar composition, moisture content, and floral origin, factors known to influence nucleation and crystal growth behavior.

**Figure 2:**
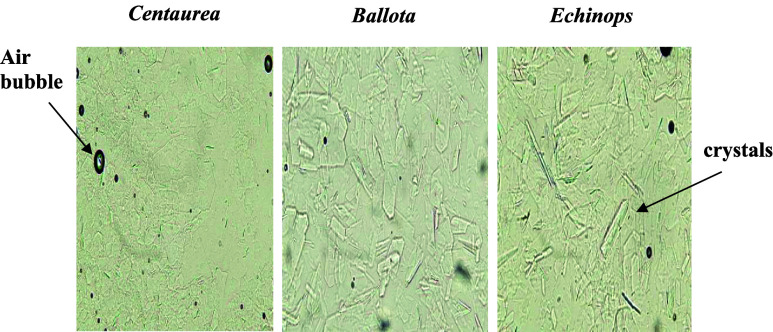
Images of crystals observation of raw honey samples under the microscope (400× magnification).

Occasional pollen grains and air bubbles were also visible, as expected for natural honeys and slide preparation, and did not interfere with crystal identification. These initial microstructural differences provide an important baseline for evaluating liquefaction efficiency and help explain the honey specific responses to microwave and conventional thermal treatments discussed in subsequent sections.

#### 3.2.2 Effect of Conventional and Microwave Treatments on Crystal Dissolution

Conventional water bath heating at 65 °C reduced crystal size and abundance in all honey samples; however, residual crystalline particles remained detectable by optical microscopy after treatment (Figure 3), indicating incomplete liquefaction under the applied time–temperature conditions. This outcome reflects the limitations of prolonged conductive heating, where heat transfer is gradual and strongly constrained by quality preservation requirements.

**Figure 3:**
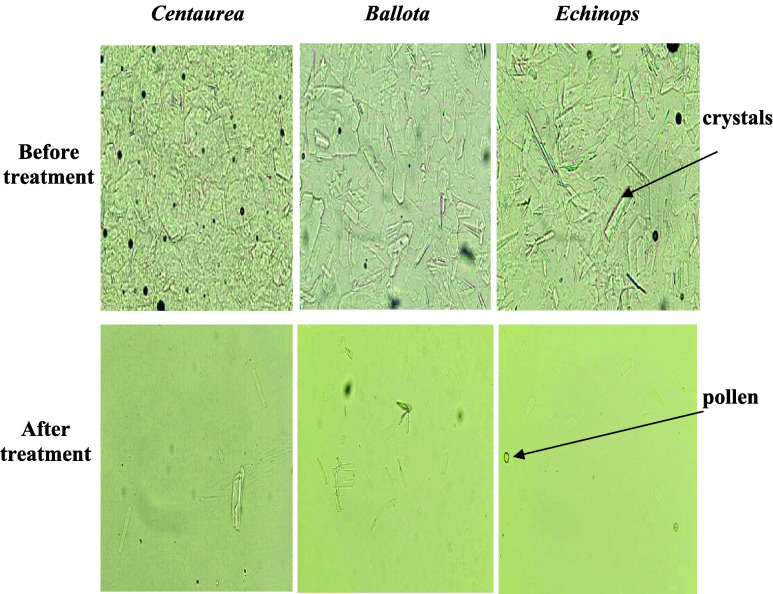
Images of crystals observation before and after conventional treatment under the microscope (400× magnification).

In contrast, microwave processing enabled the complete disappearance of visible crystals microscopically in all three honeys when optimized power–time combinations were applied (Figures 4, 5 and 6). In the context of the present study, the term “complete liquefaction” is operationally defined as the absence of visible crystalline structures under optical microscopy at the applied magnification and does not imply absolute elimination of all micro or nano scale crystalline domains. Crystals below the detection limit of the optical system may therefore remain present but unresolved under the selected imaging conditions. For each honey type, increasing microwave power consistently reduced the exposure time required for full liquefaction, demonstrating a clear inverse relationship between power level and treatment duration.

**Figure 4:**
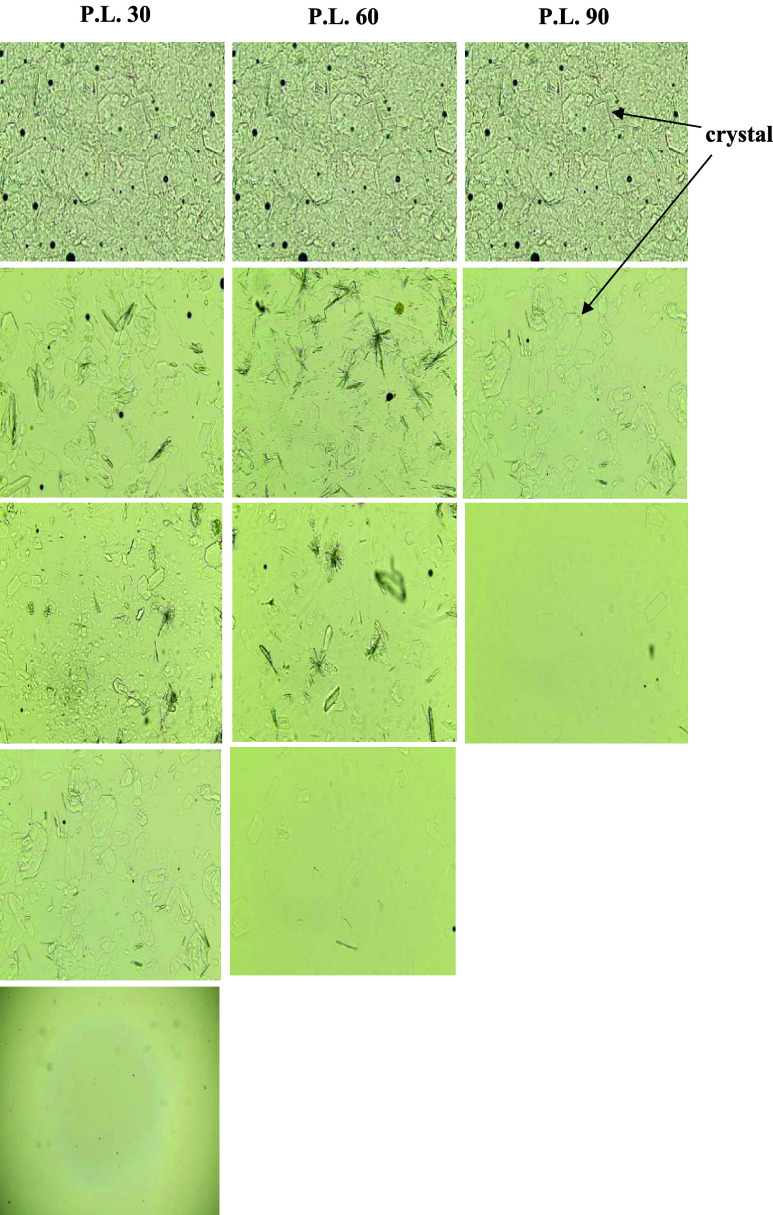
Crystals observation before and after microwave power treatments for (A) Centaurea, (B) Ballota, and (C) Echinops honeys under the microscope (400× magnification).

**Figure 5:**
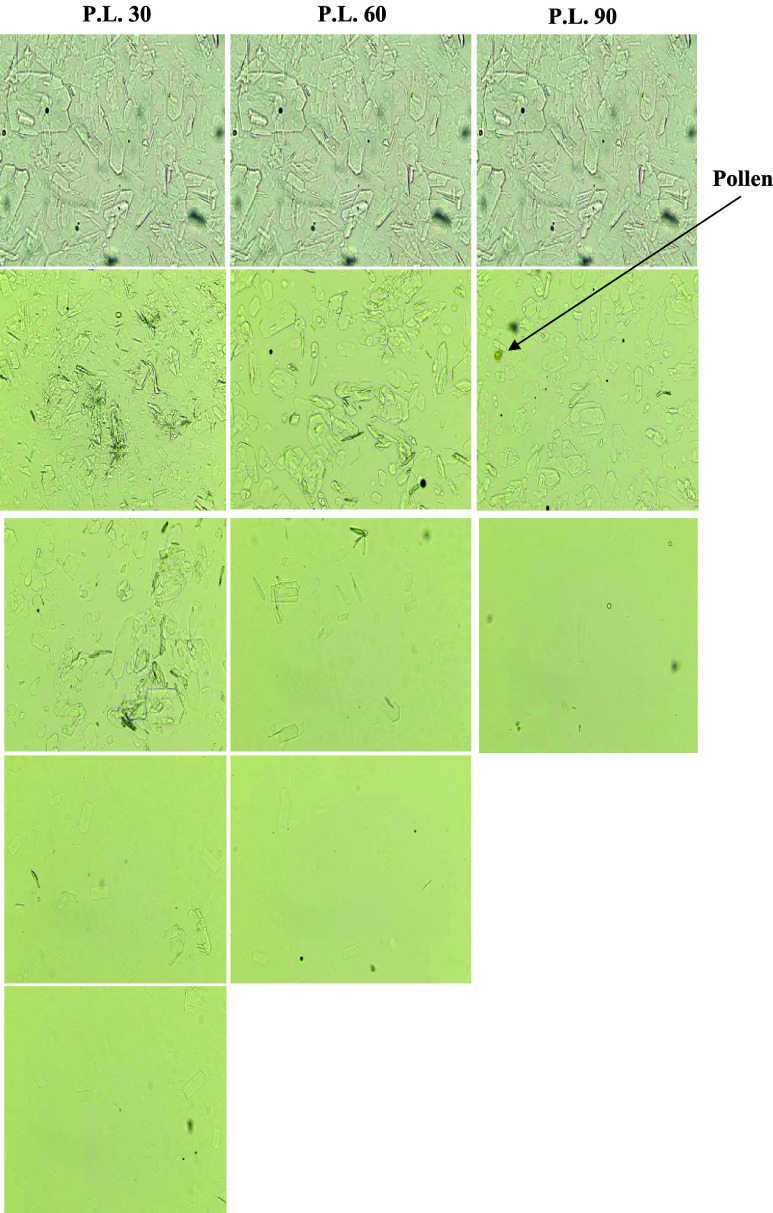
Crystals observation before and after microwave power treatments for (Ballota honey) under the microscope (400 magnification).

**Figure 6:**
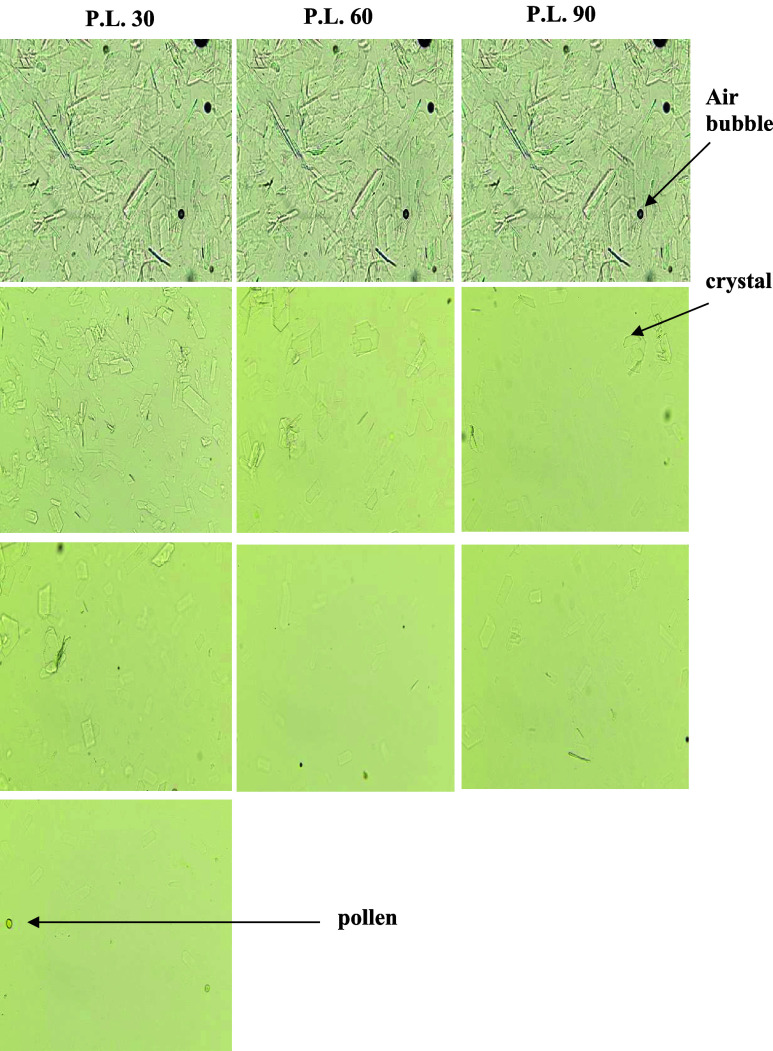
Crystals observation before and after microwave power treatments for (Echinops honey) under microscope (400 magnification).

Complete liquefaction of *Centaurea* honey was achieved at 30% power for 120s, 60% power for 90 s, or 90% power for 60 s. *Ballota* honey required 30% power for 100 s, 60% power for 75 s, or 90% power for 45 s, while *Echinops* honey was fully liquefied at 30% power for 90 s, 60% power for 60 s, or 90% power for 45 s. These results clearly demonstrate that optimal microwave liquefaction conditions are honey specific and depend on botanical origin.

The honey dependent response to microwave treatment is likely governed by compositional differences, particularly moisture content and sugar profile, which influence dielectric properties, microwave energy absorption, and heating kinetics. Honeys with higher moisture content and differing glucose–fructose ratios exhibited faster crystal dissolution under equivalent power levels. This finding supports the need for power–time optimization rather than the application of uniform microwave conditions across different honey types. Similar trends have been reported previously, where microwave treatment achieved rapid liquefaction with reduced quality deterioration compared to prolonged conventional heating.^26,27^ However, unlike earlier studies that typically examined single honey types or fixed processing conditions, the present work systematically demonstrates how botanical origin modulates microwave liquefaction efficiency within a unified experimental framework.

Crystal disappearance and liquefaction were assessed qualitatively by optical microscopy, which provides direct visual confirmation of residual crystallinity. This approach is inherently qualitative and descriptive in nature. While this method is widely applied in honey crystallization studies, it remains a qualitative assessment and does not quantify crystal size distribution, residual crystalline fraction, or the sub microscopic crystalline crystallinity. Accordingly, the conclusions regarding liquefaction efficiency should be interpreted within these methodological constraints rather than as absolute quantitative measurements of crystal elimination. Therefore, the interpretation of “complete liquefaction” should be understood within the resolution limits of optical microscopy.

Quantitative image analysis (e.g., crystal size distribution, area fraction analysis, or image based particle metrics) would provide a more objective and statistically robust evaluation of liquefaction extent. Such analyses were beyond the scope of the present study but represent an important direction for future work aimed at refining and strengthening optimization strategies, process validation, and industrial translation, including scalability assessment.

Overall, unlike prior reports that primarily confirm reduced processing time under microwave heating, the present results demonstrate that the optimal microwave regime is honey type specific, with distinct power and time thresholds required to balance complete liquefaction against enzymatic and bioactive retention.

These findings should be interpreted within the methodological framework described above, acknowledging the qualitative nature of crystal assessment and the resolution limits of optical microscopy, while emphasizing the consistent comparative trends observed across treatments.

### 3.3 Physicochemical Stability Indicators

#### 3.3.1 Moisture Content

Untreated *Centaurea*, *Ballota*, and *Echinops* honeys exhibited moisture contents of 14.6%, 15.5%, and 15.7%, respectively (Table 2). Both conventional heating and microwave treatment resulted in statistically significant reductions in moisture content across all honey types (*P* < 0.05), consistent with water evaporation during thermal exposure. Following conventional heating, moisture decreased to 13.4% (*Centaurea*), 14.1% (*Ballota*), and 13.5% (*Echinops*), while microwave treated samples showed comparable but generally less pronounced moisture losses, consistent with reduced cumulative thermal exposure under optimized regimes.

**Table 2:** Comparison of the Moisture Content, Total Soluble Solids (TSS), pH, and Electrical Conductivity (EC) of Raw Honeys (Control) and Crystallized Honeys Treated with Conventional Heating at 65 °C (C65) and Microwave Treatment at Power Levels of 30 (M30), 60 (M60), and 90 (M90)^a^

treatments	moisture (%)	TSS (°Brix)	pH	EC (mS/cm)
*C. iberica*				
Control	14.60 ± 0.12^a^	84.20 ± 0.40^a^	4.9 ± 0.01^a^	0.34 ± 0.00^b^
C65°	13.43 ± 0.15^d^	83.97 ± 0.21^ab^	4.4 ± 0.031^d^	0.36 ± 0.00^a^
M30	14.30 ± 0.20^bc^	84.43 ± 0.25^a^	4.6 ± 0.02^c^	0.34 ± 0.01^b^
M60	14.23 ± 0.06^c^	83.40 ± 0.20^c^	4.8 ± 0.04^b^	0.34 ± 0.01^b^
M90	14.50 ± 0.00^e^	83.57 ± 0.32^bc^	4.9 ± 0.11^a^	0.34 ± 0.01^b^
*B. undulate*				
Control	15.50 ± 0.15^a^	83.40 ± 0.16^a^	5.5 ± 0.01^a^	0.37 ± 0.00^b^
C65°	14.13 ± 0.25^b^	83.17 ± 0.31^b^	4.8 ± 0.01^d^	0.38 ± 0.01^a^
M30	13.07 ± 0.15^d^	83.90 ± 0.20^a^	5.3 ± 0.01^b^	0.38 ± 0.01^a^
M60	13.37 ± 0.21^cd^	83.40 ± 0.26^b^	5.2 ± 0.06^c^	0.37 ± 0.00^b^
M90	13.40 ± 0.10^c^	84.07 ± 0.32^a^	5.0 ± 0.10^d^	0.37 ± 0.01^b^
*E. polycerus*				
Control	15.70 ± 0.17^a^	84.00 ± 1.00^a^	5.3 ± 0.01^a^	0.42 ± 0.00^c^
C65°	13.30 ± 0.36^b^	83.57 ± 0.29^a^	4.8 ± 0.06^c^	0.42 ± 0.01^c^
M30	13.57 ± 0.15^b^	83.57 ± 0.35^a^	5.0 ± 0.09^b^	0.42 ± 0.01^c^
M60	13.10 ± 0.17^b^	83.27 ± 0.15^a^	5.1 ± 0.09^b^	0.44 ± 0.01^b^
M90	13.20 ± 0.36^b^	83.70 ± 0.46^a^	5.3 ± 0.03^a^	0.46 ± 0.00^a^

a Data presented as means ± standard deviation (*n* = 3). Means of the same type of honey within the column followed by the same letter are not significantly different (*P* > 0.05).

Although these differences were statistically significant, the absolute magnitude of moisture reduction (approximately 1–2%) is relatively small in practical terms. From a technological perspective, the observed reductions in moisture content were modest and remained within ranges that do not adversely affect honey handling, stability, or consumer acceptance. Accordingly, such variations are unlikely to meaningfully influence viscosity, microbial stability, or shelf life under standard commercial storage conditions. These findings align with reports indicating that thermal processing may reduce honey moisture depending on treatment intensity and configuration.^27^

Importantly, all measured moisture values remained well below the maximum limit of 20% specified by international standards and national regulations, confirming that neither conventional nor microwave liquefaction compromised regulatory compliance or marketability.^28^

#### 3.3.2 Total Soluble Solids (TTS)

Initial TSS values were 84.2, 83.4, and 84.0 °Brix for *Centaurea*, *Ballota*, and *Echinops* honeys, respectively (Table 2). Conventional heating and microwave treatment resulted in statistically significant changes in TSS for *Centaurea* and *Ballota* honeys (*P* < 0.05), reflecting changes in water fraction associated with moisture loss during thermal exposure.^29^

Although statistically significant in certain cases, the absolute differences in TSS values between untreated and treated samples were minimal. In contrast, TSS values of *Echinops* honey were not significantly altered by either treatment (*P* > 0.05), suggesting greater compositional stability of this honey matrix under the applied liquefaction conditions.

From a practical standpoint, the magnitude of TSS variation observed across treatments was small and remained within the typical range reported for high quality commercial honeys. Therefore, these changes are unlikely to represent technologically meaningful alterations in product concentration, sweetness perception, viscosity, or consumer acceptance. These findings indicate that optimized microwave processing achieves rapid liquefaction without inducing concentration effects of industrial relevance, particularly when compared with prolonged conventional heating.

#### 3.3.3 pH

Untreated honeys exhibited pH values of 4.9 (*Centaurea*), 5.5 (*Ballota*), and 5.3 (*Echinops*) (Table 2). Both conventional and microwave treatments resulted in statistically significant pH reductions (*P* < 0.05), with conventional heating producing the greatest decline. Microwave treated samples showed pH decreases that were dependent on the optimized power time regime for each honey type, reflecting differences in thermal exposure.

The observed reductions are consistent with temperature dependent shifts in hydrogen ion activity and with the release or transformation of organic acids during heating.^30–32^ Although statistically significant, the absolute pH reductions were small across all treatments and honey types, and final pH values remained within the typical acidic range characteristic of natural honeys. From a technological and microbiological perspective, these modest pH shifts are unlikely to negatively affect honey stability, safety, or sensory quality, nor do they compromise compliance with quality standards. Accordingly, while statistically significant, the observed pH variations are not considered industrially consequential under normal commercial handling and storage conditions. Compared with conventional heating, optimized microwave liquefaction achieved effective decrystallization while limiting pH alteration, supporting its suitability as a milder processing alternative.

#### 3.3.4 Electrical Conductivity (EC)

Electrical conductivity of untreated honeys ranged from 0.34 to 0.42 mS/cm (Table 2), consistent with values expected for blossom honeys. Conventional heating caused small but significant increases in EC for *Centaurea* and *Ballota* honey (*P* < 0.05), whereas *Echinops* honey showed minimal change.

Under microwave treatment, EC values of *Centaurea* and *Ballota* remained largely stable across applied power–time regimes, while *Echinops* honey exhibited a gradual increase with increasing microwave power (0.42–0.46 mS/cm). Although statistically significant in certain cases, the absolute variation in EC was limited and remained well within internationally accepted ranges for unifloral honeys.

These modest EC variations likely reflect concentration effects associated with minor moisture loss and/or increased mobility of ionic constituents during heating, rather than substantive changes in mineral composition or honey authenticity. From a technological perspective, the observed EC shifts are unlikely to affect honey classification, quality perception, or regulatory compliance. Accordingly, while statistically significant, the EC changes observed do not represent industrially meaningful alterations in mineral related quality attributes.

Particularly, microwave processing resulted in more stable EC values compared with conventional heating, further supporting its suitability as a controlled liquefaction method with minimal impact on mineral related quality indicators.

#### 3.3.5 Color (Pfund Scale)

All untreated honeys were classified as light amber according to the Pfund scale (Table 3). Conventional heating and microwave processing significantly increased color intensity in *Ballota* and *Echinops* honeys (light amber to amber; *P* < 0.05), consistent with thermally induced browning reactions, including Maillard type pathways involving sugars and amino acids.^27^ Although statistically significant, the shift in Pfund classification corresponded to a moderate a moderate transition between adjacent commercial color categories rather than excessive darkening. The resulting color values remained within commercially acceptable ranges for these honey types, indicating that thermal treatment did not lead to undesirable visual quality deterioration. Such color shifts are commonly observed during honey heating and are not necessarily indicative of quality loss when properly controlled.

**Table 3:** Comparison of Color Measurement of Raw Honeys (Control) and Crystallized Honeys Treated with Conventional Heating at 65 °C (C65) and Microwave Treatment at Power Levels of 30 (M30), 60 (M60), and 90 (M90)^a^

treatments	A_560_ × 3.150	USDA color standard designations
*C. iberica*		
Control	0.63 ± 0.002^a^	Light amber
C65	0.47 ± 0.002^e^	Extra light amber
M30	0.51 ± 0.003^c^	Extra light amber
M60	0.49 ± 0.004^d^	Extra light amber
M90	0.53 ± 0.005^b^	Extra light amber
*B. undulate*		
Control	0.81 ± 0.001^d^	Light amber
C65	1.55 ± 0.002^c^	Amber
M30	1.55 ± 0.005^c^	Amber
M60	1.57 ± 0.003^b^	Amber
M90	1.60 ± 0.002^a^	Amber
*E. polycerus*		
Control	0.92 ± 0.002^d^	Light amber
C65	1.58 ± 0.004^c^	Amber
M30	1.58 ± 0.002^c^	Amber
M60	1.93 ± 0.002^b^	Amber
M90	1.96 ± 0.002^a^	Amber

a Data presented as means ± standard deviation (*n* = 3). Means of the same type of honey within the column followed by the same letter are not significantly different (*P* > 0.05).

In contrast, *Centaurea* honey became lighter following treatment (light amber to extra light amber). This atypical response suggests degradation or transformation of specific pigments or phenolic complexes characteristic of *Centaurea* honey, rather than Maillard driven browning. Similar honey specific color responses have been attributed to differences in floral derived compounds and their thermal stability. Although statistically significant, this lightening effect also remained within standard commercial Pfund classifications and is unlikely to negatively influence consumer perception or market grading.

Overall, these findings highlight that color response to liquefaction is strongly dependent on botanical origin and that optimized microwave processing can achieve effective liquefaction without inducing excessive or commercially undesirable color changes, supporting its suitability for maintaining consumer acceptable appearance.

### 3.4 Hydroxymethylfurfural (HMF) Formation

Hydroxymethylfurfural (HMF) content increased significantly (*P* < 0.05) in all honeys following both conventional and microwave treatments (Table 4), reflecting thermally induced sugar degradation pathways, particularly fructose dehydration and Maillard type reactions under acidic conditions.^33^ HMF is widely recognized as a sensitive indicator of thermal load in honey and is therefore central to assessing processing severity and regulatory compliance.

**Table 4:** Comparison of Hydroxymethylfurfural (HMF), Total Phenolics Content (TPC), and Antioxidant Activity of Raw Honeys (Control) and Crystallized Honeys Treated with Conventional Heating at 65 °C (C65) and Microwave Treatment at Power Levels of 30 (M30), 60 (M60), and 90 (M90)^a^

treatment	HMF (mg/kg)	TPC (mg GAE/100 g)	antioxidant activity (% inhibition)
C. iberica			
Control	6.91 ± 0.26^e^	40.43 ± 0.42^a^	38.33 ± 1.04^a^
C65	21.93 ± 0.64^a^	33.53 ± 0.80^b^	28.90 ± 0.26^d^
M30	8.92 ± 0.16^c^	31.07 ± 1.02^c^	34.90 ± 0.87^c^
M60	8.32 ± 0.01^d^	40.07 ± 0.55^a^	31.20 ± 0.26^b^
M90	12.09 ± 0.17^b^	28.53 ± 0.55^d^	30.07 ± 1.27^cd^
*B. undulate*			
Control	7.01 ± 0.09^d^	44.80 ± 0.36^a^	44.50 ± 0.95^a^
C65	20.87 ± 0.43^a^	36.57 ± 1.77^b^	28.80 ± 0.58^e^
M30	8.07 ± 0.08^c^	38.17 ± 0.25^b^	35.80 ± 0.36^c^
M60	7.35 ± 0.12^d^	44.00 ± 0.26^a^	40.03 ± 0.20^b^
M90	13.50 ± 0.27^b^	32.30 ± 0.78^c^	31.20 ± 0.49^d^
*E. polycerus*			
Control	7.95 ± 0.14^d^	47.17 ± 0.71^a^	38.57 ± 0.67^a^
C65	18.99 ± 0.19^a^	36.70 ± 0.35^d^	27.13 ± 0.32^d^
M30	8.96 ± 0.19^c^	39.20 ± 0.85^c^	30.33 ± 0.78^c^
M60	8.94 ± 0.16^c^	40.83 ± 0.80^b^	32.80 ± 0.30^b^
M90	12.53 ± 0.29^b^	36.83 ± 0.25^d^	27.03 ± 0.31^d^

a Data presented as means ± standard deviation (*n* = 3). Means of the same type of honey within the column followed by the same letter are not significantly different (*P* > 0.05).

Conventional heating at 65 °C for 25 min resulted in the highest HMF accumulation, reaching 21.93 mg/kg in *Centaurea*, 20.78 mg/kg in *Ballota*, and 18.99 mg/kg in *Echinops* honey. In contrast, microwave treated samples consistently exhibited lower HMF levels, with the magnitude of increase dependent on the optimized power and time regime applied. For *Centaurea* honey, HMF values ranged from 8.32 to 12.09 mg/kg across microwave treatments; *Ballota* honey ranged from 7.35 to 13.50 mg/kg; and *Echinops* honey from 8.94 to 12.53 mg/kg (Table 4). These results demonstrate a clear time and temperature dependence of HMF formation, whereby prolonged exposure during conventional heating promoted greater HMF accumulation than the shorter, optimized microwave regimes. Although microwave heating is known to produce non uniform temperature fields, the substantially reduced processing times appear to limit cumulative thermal damage, resulting in lower net HMF formation compared with conventional water-bath heating. This trend is consistent with previous studies reporting substantially higher HMF levels following extended thermal treatments relative to microwave processing.^27,34–36^ While the increases in HMF were statistically significant, the absolute concentrations observed under both treatments remained well below the maximum limit of 40 mg/kg specified by Codex standards^28^ and within ranges commonly encountered in commercially processed honeys. Accordingly, although statistically significant, these HMF elevations do not represent regulatory noncompliance or immediate industrial concern under the applied processing conditions.

From a technological perspective, these findings indicate that optimized microwave-assisted liquefaction can achieve rapid and complete decrystallization while minimizing HMF accumulation, thereby offering a practical advantage over prolonged conventional heating in terms of quality preservation and regulatory safety margins.

### 3.5 Bioactive Related Quality Attributes: Total Phenolic Content and Antioxidant Activity

#### 3.5.1 Total Phenolic Content (TPC)

Untreated honeys exhibited total phenolic contents of 40.43, 44.80, and 47.17 mg GAE/100 g for *Centaurea*, *Ballota*, and *Echinops* honeys, respectively (Table 4), values that fall within the range reported for comparable unifloral honeys and reflect inherent differences linked to botanical origin. Conventional heating caused a significant reduction in TPC across all honey types (*P* < 0.05), decreasing to 33.53–36.70 mg GAE/100 g, reflecting the susceptibility of phenolic compounds to prolonged thermal exposure and oxidative degradation.

Microwave assisted liquefaction also reduced TPC; however, the magnitude of loss depended on the applied power and time regime. Notably, intermediate microwave power (PL60) consistently preserved higher phenolic levels than both lower and higher power treatments, yielding values of 40.07, 44.00, and 40.83 mg GAE/100 g for *Centaurea*, *Ballota*, and *Echinops* honeys, respectively (Table 4). This non linear response highlights the importance of power and time optimization, as excessive exposure or overly intense microwave conditions may negate the potential benefits of rapid heating.

The observed reductions are consistent with thermal degradation and oxidation of phenolic compounds during processing. Phenolics may undergo structural modification, polymerization, or interaction with Maillard reaction intermediates under heat, leading to reduced measurable content. Although contrasting trends have been reported in the literature, including apparent increases in TPC under certain microwave conditions, such discrepancies are generally attributed to differences in botanical origin, matrix composition, analytical release of bound phenolics, and treatment severity.^37–42^ In the present study, cumulative thermal exposure, rather than heating modality per se, was identified as the dominant determinant of phenolic loss, with optimized microwave treatment offering a measurable advantage over prolonged conventional heating.

Although the reductions in TPC were statistically significant, the absolute changes observed under optimized microwave conditions were relatively modest compared with conventional heating and remained within ranges reported for commercially available honeys of similar floral origin. From a practical perspective, these findings suggest that microwave assisted liquefaction, when carefully optimized, can better preserve phenolic compounds that contribute to honey’s functional value, without compromising processing efficiency or regulatory compliance. Therefore, while statistically significant, the phenolic losses under optimized microwave treatment are unlikely to diminish the perceived functional or market value of the product in typical commercial contexts.

#### 3.5.2 Antioxidant Activity (DPPH Radical Scavenging)

Antioxidant activity of untreated honeys, expressed as DPPH radical scavenging, was 33.33% for *Centaurea*, 44.50% for *Ballota*, and 38.57% for *Echinops* honey (Table 4). Conventional heating resulted in the greatest reduction in antioxidant activity (*P* < 0.05), decreasing values to 27.13–29.80%, consistent with thermal degradation and structural modification of antioxidant related constituents.

Microwave assisted liquefaction also reduced antioxidant activity in all honeys, but the magnitude of loss was consistently lower than that observed under conventional heating, indicating improved preservation under optimized microwave conditions. The extent of reduction varied with honey type and processing regime, reflecting differences in phenolic composition, matrix interactions, and thermal sensitivity among botanical origins.

Importantly, although statistically significant, the observed reductions in antioxidant activity under optimized microwave regimes were modest in magnitude and remained within ranges reported for commercially acceptable processed honeys, suggesting limited practical impact on functional quality. From a technological perspective, these statistically significant changes are unlikely to materially affect overall antioxidant classification, product positioning, or perceived health related value in routine commercial contexts. These findings align with previous studies indicating that antioxidant activity reflects a balance between degradation of native polyphenols and the formation of heat induced reaction products during processing.^12^

### 3.6 Enzymatic Quality Indicators: Diastase and Invertase Activities

#### 3.6.1 Diastase Activity

Diastase numbers (DN) of untreated honeys ranged from 13.24 to 14.98, confirming good initial enzymatic quality (Table 5). Conventional heating caused a pronounced reduction in diastase activity across all honey types (*P* < 0.05), decreasing DN to 7.24 (*Centaurea*), 8.00 (*Ballota*), and 8.11 (*Echinops*). In the case of *Centaurea* honey, values fell below the commonly accepted minimum limit of DN = 8, indicating substantial enzymatic inactivation under prolonged thermal exposure.

**Table 5:** Comparison of Diastase and Invertase Numbers of Raw Honeys (Control) and Crystallized Honeys Treated with Conventional Heating at 65 °C (C65) and Microwave Treatment at Power Levels of 30 (M30), 60 (M60), and 90 (M90)^a^

treatment	diastase number	invertase number
*C. iberica*		
Control	13.24 ± 0.11^a^	18.20 ± 0.36^a^
C65	7.24 ± 0.49^d^	6.47 ± 0.31^e^
M30	11.70 ± 0.18^b^	10.17 ± 0.06^d^
M60	12.00 ± 0.26^b^	14.80 ± 0.10^b^
M90	8.83 ± 0.21^c^	10.63 ± 0.15^c^
*B. undulate*		
Control	14.48 ± 0.17^a^	20.33 ± 0.25^a^
C65	8.00 ± 0.26^d^	5.57 ± 0.31^e^
M30	11.70 ± 0.40^c^	11.93 ± 0.15^c^
M60	12.83 ± 0.21^b^	13.60 ± 0.10^b^
M90	8.17 ± 0.31^d^	9.80 ± 0.10^d^
*E. polycerus*		
Control	14.98 ± 0.19^a^	22.50 ± 0.36^a^
C65	8.11 ± 0.47^d^	7.50 ± 0.36^e^
M30	9.17 ± 0.25^c^	12.53 ± 0.21^c^
M60	11.93 ± 0.25^b^	14.57 ± 0.12^b^
M90	8.77 ± 0.06^c^	9.97 ± 0.12^d^

a Data presented as means ± standard deviation (*n* = 3). Means of the same type of honey within the column followed by the same letter are not significantly different (*P* > 0.05).

Microwave assisted liquefaction also reduced diastase activity relative to untreated controls; however, enzyme losses were consistently lower than those observed under conventional heating, reflecting reduced cumulative thermal stress. Under optimized microwave regimes, DN values remained above or close to the regulatory minimum for all honey types, with the highest retention observed at moderate power and time combinations (Table 5). For example, PL60 treatments preserved DN values of 12.00 (*Centaurea*), 13.60 (*Ballota*), and 11.93 (*Echinops*), substantially higher than those obtained by conventional heating.

While reductions in DN were statistically significant, their technological relevance differed between treatments. In the case of conventional heating, the decline below the regulatory threshold for Centaurea honey represents a practically meaningful quality loss with potential implications for compliance and market grading. In contrast, under optimized microwave regimes, although statistically significant, the remaining DN values were maintained within or near acceptable limits, thereby preserving enzymatic quality in a manner unlikely to compromise regulatory conformity or commercial classification.

These results demonstrate that diastase retention is governed primarily by cumulative thermal exposure rather than heating modality per se, and that microwave processing, when appropriately optimized, can achieve rapid liquefaction while maintaining enzymatic quality within acceptable regulatory limits. Similar inverse relationships between heating duration and diastase activity have been widely reported, confirming the suitability of diastase as a sensitive marker of honey processing severity.^11,13,36,43,44^

#### 3.6.2 Invertase Activity

Invertase activity was consistently more sensitive to thermal processing than diastase activity, confirming its suitability as a stringent indicator of processing severity. Untreated honeys exhibited invertase numbers (IN) of 18.20, 20.33, and 22.50 for *Centaurea*, *Ballota*, and *Echinops* honeys, respectively (Table 5), reflecting good initial enzymatic quality. Conventional heating caused pronounced and uniform losses in invertase activity across all honey types (*P* < 0.05), reducing IN to 5.57–7.50, well below the recommended minimum value of 10. These results indicate substantial enzyme denaturation under prolonged conventional thermal exposure, despite the use of temperatures commonly applied in industrial practice.

Microwave assisted liquefaction also resulted in significant reductions in invertase activity; however, enzyme retention was strongly dependent on the applied power and time regime rather than on microwave heating per se. Intermediate microwave conditions (e.g., PL60 with optimized exposure times) preserved invertase activity more effectively than both conventional heating and high power microwave treatments. In contrast, at the highest microwave power (PL90), invertase values approached or slightly fell below the minimum recommended threshold, even under optimized exposure times, highlighting the critical importance of power and time optimization for thermolabile enzymes.

Although reductions in invertase activity were statistically significant across treatments, their technological implications varied substantially. Under conventional heating, the marked decline below the recommended minimum threshold represents a practically meaningful loss of enzymatic quality with potential consequences for regulatory grading and commercial classification. In contrast, under optimized intermediate microwave regimes, despite statistically significant decreases, invertase levels were better preserved and, in most cases, remained closer to acceptable standards, thereby mitigating industrial impact.

The pronounced heat sensitivity of invertase to heat has been well documented and reflects its lower thermal stability relative to diastase.^45,46^ Importantly, the present results demonstrate that optimized microwave liquefaction can reduce cumulative thermal damage to invertase compared with conventional heating, but only within a defined processing window. This finding highlights that microwave processing is not inherently non destructive and must be carefully controlled to balance liquefaction efficiency with enzymatic quality preservation.

### 3.7 Sugars Profile

The sugar composition of untreated honeys varied modestly with botanical origin (Table 6). *Centaurea* honey contained fructose, glucose, sucrose, and maltose at 40.33, 35.23, 2.93, 0.93 g/100 g, respectively. *Ballota* honey exhibited slightly higher glucose and maltose contents (37.50 and 1.99 g/100 g), while *Echinops* honey showed the highest glucose proportion (40.10 g/100 g). These differences reflect expected botanical influences on nectar composition and crystallization behavior.

**Table 6:** Comparison of Fructose, Glucose, Sucrose, and Maltose Contents (in %, w/w) in Raw Honeys (Control) and Crystallized Honeys Treated with Conventional Heating at 65 °C (C65) and Microwave Treatment at Power Levels of 30 (M30), 60 (M60), and 90 (M90)^a^

treatments	fructose	glucose	sucrose	maltose
*C. iberica*				
Control	40.33 ± 0.71^a^	35.23 ± 0.40^a^	2.93 ± 0.21^a^	0.93 ± 0.06^a^
C65	36.30 ± 0.25^c^	28.17 ± 0.7 ^d^	2.83 ± 0.06^a^	0.68 ± 0.00^c^
M30	38.13 ± 0.60^b^	32.67 ± 0.76^b^	2.67 ± 0.32^a^	0.92 ± 0.05^a^
M60	40.17 ± 0.35^a^	34.00 ± 0.50^a^	2.70 ± 0.26^a^	0.75 ± 0.05^c^
M90	39.47 ± 0.55^b^	29.83 ± 1.04^c^	2.93 ± 0.15^a^	0.70 ± 0.01^c^
*B. undulate*				
Control	40.73 ± 0.72^a^	37.50 ± 0.56^a^	3.10 ± 0.10^ab^	1.99 ± 0.06^a^
C65	38.07 ± 0.20^bc^	29.67 ± 0.80^cd^	3.23 ± 0.15^a^	1.03 ± 0.15^b^
M30	37.83 ± 0.80^c^	28.67 ± 1.04^d^	2.83 ± 0.15^bc^	0.78 ± 0.03^c^
M60	39.10 ± 0.56 ^b^	34.50 ± 0.50^b^	3.00 ± 0.10^ab^	0.63 ± 0.03^d^
M90	38.80 ± 0.70^bc^	30.50 ± 0.50^c^	2.63 ± 0.35^c^	0.58 ± 0.02^d^
*E. polycerus*				
Control	39.97 ± 1.05^a^	40.17 ± 1.26^a^	3.10 ± 0.20^a^	1.03 ± 0.16^a^
C65	35.50 ± 0.26^b^	25.67 ± 0.58^e^	3.00 ± 0.10^a^	0.65 ± 0.05^c^
M30	35.13 ± 0.25^bc^	31.33 ± 0.58^c^	2.90 ± 0.10^a^	0.57 ± 0.08^c^
M60	38.93 ± 0.50^a^	35.50 ± 0.50^b^	2.87 ± 0.15^a^	0.83 ± 0.06^b^
M90	38.70 ± 0.50^ab^	29.00 ± 0.87^d^	2.57 ± 0.15^b^	0.70 ± 0.0^bc^

a Data presented as means ± standard deviation (*n* = 3). Means of the same type of honey within the column followed by the same letter are not significantly different (*P* > 0.05).

Both conventional heating and microwave assisted liquefaction resulted in statistically significantly reduction in fructose, glucose, and maltose contents across all honey types (*P* < 0.05), whereas sucrose levels were not significantly affected (*P* > 0.05). The magnitude of these reductions was consistently greater under prolonged conventional heating, while optimized microwave treatments produced smaller decreases, consistent with reduced cumulative thermal exposure.

Although statistically significant, the absolute reductions in individual sugars were relatively small in magnitude. In practical processing terms, such changes do not materially alter the fundamental carbohydrate composition of honey but rather reflect expected thermal participation of reducing sugars in secondary reactions.

The observed reductions are consistent with the thermal sensitivity of reducing sugars and their participation in dehydration, Maillard, and caramelization reactions, which are also linked to hydroxymethylfurfural formation.^47,48^ Literature reports on the effect of microwave processing on honey sugars remain variable, reflecting differences in honey matrix composition, power intensity, and exposure duration.^44,47–55^ In the present study, sugar degradation was driven primarily by processing severity rather than heating modality alone.

Importantly, despite statistical significance, the combined fructose and glucose contents of all treated honeys remained well above the minimum limits specified by Codex Alimentarius and national honey standards (≥60 g/100 g).^28^ Therefore, while analytically significant, these compositional shifts are unlikely to have meaningful implications for regulatory compliance, product classification, sweetness perception, or consumer acceptability under the applied liquefaction conditions. This distinction between statistical change and technological relevance is critical when evaluating processing strategies.

Collectively, these findings indicate that optimized microwave liquefaction achieves effective decrystallization while preserving the fundamental carbohydrate profile of honey, whereas prolonged conventional heating leads to greater sugar losses without conferring additional regulatory or functional benefit.

While microwave assisted liquefaction demonstrated clear advantages in processing speed and quality preservation under laboratory conditions, several practical considerations should be acknowledged. Microwave heating is known to be susceptible to uneven energy distribution and localized hot spots, particularly in viscous, high sugar matrices such as honey, which may affect thermal uniformity if not carefully controlled. In addition, scalability and energy efficiency remain critical factors for industrial adoption, as processing performance can vary with sample volume, container geometry, and equipment design. Potential effects on sensory attributes, including flavor and aroma, were not evaluated in the present study and warrant further investigation. Addressing these aspects through continuous temperature monitoring, pilot-scale validation, and sensory assessment will be essential for translating optimized microwave liquefaction protocols into robust industrial applications.

Overall, the present study was conducted using three monofloral honeys obtained from a single geographic region and producer, which may limit direct extrapolation of the findings to all commercial honeys. Honey composition, crystallization behavior, and thermal sensitivity are known to vary with botanical origin, geographic provenance, and moisture content. Accordingly, the optimized microwave power and time regimes identified here should be interpreted as honey type specific reference conditions rather than universally applicable parameters. Nevertheless, by employing a controlled experimental design and benchmarking microwave processing directly against conventional heating, this study provides a reproducible framework that can be applied to other honey types to guide future optimization and scale up studies.

Across all evaluated quality indicators, including moisture, TSS, pH, electrical conductivity, color, HMF formation, phenolic content, antioxidant activity, enzymatic activity, and sugar composition, a consistent pattern emerged in which treatment severity, rather than heating modality alone, governed quality outcomes. The comparative differences between microwave and conventional liquefaction can be largely interpreted through the concept of cumulative thermal load, reflecting the combined influence of temperature intensity and exposure duration. Prolonged conductive heating resulted in greater cumulative thermal stress, promoting higher HMF formation, enzyme inactivation, phenolic degradation, and sugar participation in secondary reactions. In contrast, optimized microwave treatments achieved effective crystal dissolution within substantially shorter exposure times, thereby limiting cumulative thermal damage despite localized energy absorption characteristics. This integrative interpretation provides a unifying mechanistic framework for understanding the trends observed across multiple parameters and highlights the importance of power–time optimization rather than heating technology alone.

## 4 Conclusions

This study demonstrates that microwave assisted liquefaction, when systematically optimized, constitutes a rapid and controllable alternative to conventional thermal treatment for crystallized honey, with clear advantages in processing efficiency and quality preservation. Conventional water bath heating reduced crystal size but did not consistently achieve complete liquefaction and was associated with greater cumulative thermal damage, particularly to heat sensitive quality indicators. In contrast, microwave processing enabled complete crystal dissolution in all tested honeys when power and time regimes were optimized according to botanical origin, highlighting the importance of honey specific process design.

Across *Centaurea*, *Ballota*, and *Echinops* honeys, optimized microwave treatments achieved liquefaction in substantially shorter times, resulting in lower hydroxymethylfurfural formation and superior retention of enzymatic activity (diastase and invertase) compared with prolonged conventional heating. Although reductions in total phenolic content and antioxidant activity were observed under both processing methods, losses were consistently less pronounced under moderate, optimized microwave conditions, indicating that cumulative thermal exposure, rather than heating modality per se, is the primary driver of quality degradation. Importantly, all treated samples complied with international regulatory limits for moisture, HMF, enzymatic activity, and sugar composition, confirming the technological feasibility of both approaches within quality standards.

From an application and technology transfer perspective, these findings indicate that microwave liquefaction can offer meaningful advantages for honey processors, including reduced processing time, improved preservation of quality sensitive constituents, and enhanced process controllability. However, the results also demonstrate that microwave processing is not universally benign and requires careful power and time optimization tailored to honey botanical origin to avoid enzymatic losses at high power levels.

While the present study is limited to three monofloral honeys from a single geographic region, certain findings are likely generalizable beyond the tested samples, whereas others are inherently honey specific. The overarching observation that cumulative thermal load governs physicochemical, enzymatic, and bioactive changes, and that reduced exposure time can mitigate quality degradation, is expected to apply broadly across honey types. In contrast, the precise power–time combinations required for complete liquefaction, as well as the magnitude of enzymatic or bioactive losses, are dependent on botanical origin, sugar composition, moisture content, and matrix specific dielectric properties. Accordingly, the optimized conditions reported here should be interpreted as honey specific reference parameters rather than universally transferable processing settings. Nevertheless, the integrative framework presented provides a practical basis for extending microwave liquefaction strategies to other honeys through targeted optimization and future scale up and energy efficiency assessments.

Overall, this work supports microwave assisted liquefaction as a viable and scientifically grounded alternative to prolonged conventional heating, offering a balanced approach that combines effective decrystallization with preservation of physicochemical and biochemical quality when appropriately optimized. Despite these advantages, further work is required to evaluate scalability, energy efficiency, thermal uniformity, and sensory impacts under industrial processing conditions, which will be essential for broader commercial implementation.
